# Probing Corticospinal Recruitment Patterns and Functional Synergies with Transcranial Magnetic Stimulation

**DOI:** 10.3389/fncel.2016.00175

**Published:** 2016-07-05

**Authors:** James Mathew, Angelika Kübler, Robert Bauer, Alireza Gharabaghi

**Affiliations:** ^1^Division of Functional and Restorative Neurosurgery, Eberhard Karls University TübingenTübingen, Germany; ^2^Centre for Integrative Neuroscience, Eberhard Karls University TübingenTübingen, Germany

**Keywords:** motor-evoked potential, stimulus–response curve, cortical motor map, forearm muscles, abnormal synergies, plasticity, stroke, neurorehabilitation

## Abstract

**Background:** On the one hand, stimulating the motor cortex at *different spots* may activate the same muscle and result in a muscle-specific cortical map. Maps of different muscles, which are functionally coupled, may present with a large overlap but may also show a relevant variability. On the other hand, stimulation of the motor cortex at *one spot* with different stimulation intensities results in a characteristic input–output (IO) curve for one specific muscle but may simultaneously also activate different, functionally coupled muscles. A comparison of the cortical map overlap of synergistic muscles and their IO curves has not yet been carried out.

**Objective:** The aim of this study was to probe functional synergies of forearm muscles with transcranial magnetic stimulation by harnessing the convergence and divergence of the corticospinal output.

**Methods:** We acquired bihemispheric cortical maps and IO curves of the extensor carpi ulnaris, extensor carpi radialis, and extensor digitorum communis muscles by subjecting 11 healthy subjects to both monophasic and biphasic pulse waveforms.

**Results:** The degree of synergy between pairs of forearm muscles was captured by the overlap of the cortical motor maps and the respective IO curves which were influenced by the pulse waveform. Monophasic and biphasic stimulation were particularly suitable for disentangling synergistic muscles in the right and left hemisphere, respectively.

**Conclusion:** Combining IO curves and different pulse waveforms may provide complementary information on neural circuit dynamics and corticospinal recruitment patterns of synergistic muscles and their neuroplastic modulation.

## Introduction

### Convergence and Divergence

The cortical overlap and covariation of motor-evoked potentials (MEP) can be captured by simultaneous recordings of electromyography (EMG) from different muscles during brain stimulation. Due to the neural circuit architecture and synaptic connectivity in the motor cortex, convergence and divergence occur as two parallel mechanisms. Stimulation of different cortical spots may result in activation of the same muscle (“convergence”), while stimulation of the same spot may result in the simultaneous activation of different muscles (“divergence”; Melgari et al., 2008).

However, the cortical mapping techniques for probing effective corticospinal connectivity are different in animal research and human studies with regard to their level of invasiveness and spatial accuracy. Intracortical microstimulation in animal experiments revealed that both separated areas in the primary motor cortex converge on the same muscle and that they diverge motor output from the same cortical area on separate but related muscles. These mechanisms are probably mediated via intracortical and/or intraspinal projections that interconnect different functional sites.

### Cortical Motor Maps

Transcranial magnetic stimulation (TMS)—albeit with less spatial resolution than surgical mapping techniques—has been established as a powerful alternative mapping tool for clinical and research application in humans (Rossini et al., 2015). Although convergence and divergence are also present in TMS motor maps, they show a different pattern. While microstimulation detects spatially discrete spots—characterized by MEPs of the same muscle—*intermingled* with non-responsive areas or spots characterized by another muscle (Donoghue et al., 1992), TMS reveals rather extended cortical areas related to one muscle *overlapping* with the areas related to other muscles (Devanne et al., 2006; Melgari et al., 2008). Similarly, due to the induction of a *larger electrical field*, TMS activates more muscles simultaneously than microstimulation. While the first TMS feature of *overlapping* has already been used in the past, we aim in this study to harness the second feature—the *larger electrical field*—to probe muscle synergies of the forearm.

More specifically, previous studies acquired cortical motor maps at one stimulation intensity, e.g., at 120% of the resting motor threshold, and at different spots of a predefined virtual grid covering the motor cortex. Functional associations between pairs of muscles were captured by the degree of overlap, i.e., the percentage of grid positions on which TMS elicited a MEP response in two muscles with respect to the number of grid positions on which TMS elicited a MEP in at least one of the muscles (Melgari et al., 2008). A larger overlap, e.g., within and between hand and forearm muscles, is ascribed to a functional coupling of these muscles mediated via intracortical connectivity. This cortical map overlap of muscles that are commonly used together cannot be explained by stimulus spread only, since sharp demarcations between the borders of these territories were also apparent (Devanne et al., 2006). However, more recent studies have revealed relevant variability of the spatial extent of motor maps independent of any intervention (Weiss et al., 2013), thus questioning the reliability of cortical map overlap as a marker for muscle synergies.

### A Novel Approach

In this paper, we propose an alternative approach for probing muscle synergies, i.e., by acquiring input–output (IO) curves at one cortical spot, but with different pulse waveforms. Both IO curves and pulse waveforms, i.e., monophasic or biphasic, have already been shown to provide complementary information on neural circuit dynamics and recruitment patterns of corticospinal excitability (Devanne et al., 1997; Di Lazzaro et al., 2001a,b). By combining these techniques with simultaneous EMG recordings of different muscles, we reasoned that the systematic increase of stimulation intensity could disentangle the respective neural circuitry, i.e., recruitment gain of intracortical networks, corticospinal output, and motor neuronal pools, to capture different levels of muscle synergy. We examined the forearm muscles extensor carpi ulnaris (ECU), extensor carpi radialis (ECR), and extensor digitorum communis (EDC) on account of their synergistic interaction on wrist extension as well as their separate function for wrist adduction, wrist abduction, and finger extension, respectively.

### Rationale for the Study

We hypothesized that the degree of synergy between pairs of these muscles, captured by the respective overlap of cortical motor maps, is also reflected in the similar IO curves and anticipated that the applied pulse waveform can disentangle the involved neuronal circuitry. This assumption was based on the following rationale: Due to the vicinity of the cortical representations of these three muscles we would expect no differences of their centers of gravity (CoG). In this case, any differences of the IO curves for pairs of these muscles elicited at the same stimulation spot with the same stimulation intensity would indicate the involvement of different neural circuitries. If such differences would be specific for a particular pulse waveform, this would indicate the involvement of specific intracortical networks. Furthermore, when such *different* IO curves would exist for one pair of these three muscles, a *similar* IO curve for another pair of them, when elicited at the same cortical spot, with the same stimulation intensity and the same pulse waveform, would indicate that this finding is not attributable to pure current spread but rather to the involved neural circuitry.

## Materials and Methods

### Subjects

Eleven healthy subjects (mean age, 28 ± 7.5 years, range 22–35 years, five male) with no contraindications to TMS (Rossi et al., 2009) and no history of psychiatric or neurological disease were recruited for this study. Right-handedness was confirmed by the Edinburgh Handedness Inventory (Oldfield, 1971). All subjects gave their prior written informed consent to participation in the study, which had been approved by our local ethics committee. The study conformed to the latest version of the Declaration of Helsinki. The subjects participated in four measurements in random order: monophasic stimulation of the left hemisphere, monophasic stimulation of the right hemisphere, biphasic stimulation of the left hemisphere, and biphasic stimulation of the right hemisphere. On 1 day, only one pulse wave form (monophasic or biphasic) was applied to both hemispheres. Experiments were separated by 2 days in all but one subject. The order of experiments within 1 day (right vs. left hemisphere) and the order of measurement days (monophasic vs. biphasic) were carried out in random order.

### Recordings

#### Electromyography

We used the integrated EMG device of the eXimia Navigated Brain Stimulation (NBS) system (Nexstim Inc., Finland) with 3 kHz sampling rate and band-pass filter of 10–500 Hz to record EMG activity from the left and right ECU, ECR, and EDC muscle. We placed two electrodes (Ag/AgCl Ambu Neuroline 720 wet gel surface electrodes, Ambu GmbH, Germany) on each muscle belly 2 cm apart from each other. The muscle bellies were localized with palpation during the respective movements specific to each muscle, i.e., wrist adduction, wrist abduction and finger extension.

### TMS Protocol

A navigated TMS (nTMS) stimulator (eXimia^®^, Nexstim, Helsinki, Finland) with mono- or biphasic current waveform connected to a **Figure 8** eXimia Focal Bipulse Coil was used to determine MEP IO curves. Prior to the experiment, we used a 1.5- or 3-T Siemens magnetic resonance imaging (MRI) system (Siemens AG, Germany) to obtain anatomical T1-weighted MRI sequences for each participant. Images were loaded into the eXimia NBS system for coregistration with the participant’s head. We applied either a monophasic (focal monopulse coil—5.9 cm mean winding diameter) or a biphasic single pulse coil (focal bipulse coil—5 cm mean winding diameter) and the anatomically defined “hand knob” of the primary motor cortex (M1) as the starting position. The orientation of the induced current of the stimulus in the brain was posterior–anterior for the monophasic waveform, and posterior–anterior in the first and anterior–posterior in the second phase of the biphasic waveform, respectively.

Subjects were seated in a comfortable reclining chair. We used 40% of maximum stimulator output (MSO) and the anatomically defined “hand knob” of the motor cortex as the starting intensity. Whenever there was not enough initial stimulator output to elicit MEPs of the forearm extensors, we increased the output in 5% steps. The orientation of the electric field, calculated with the individual MRI of each subject by the NBS software, was kept perpendicular to the central sulcus as a starting position, and the location with the highest MEP response for the ECU muscle was selected as the stimulation point. The highest responses for the EDC and ECR muscles were, however, in the immediate vicinity, i.e., separated by an average Euclidian distance of 3.95 and 6.85 mm from the highest MEP response of the ECU muscle, respectively. Having located the ECU hotspot by moving the coil around the hand knob, we refined and varied the orientation of the coil in steps of roughly 10° around the original orientation to determine the orientation with the highest response in this spot. Resting motor threshold (RMT) was ascertained using the relative frequency method, i.e., by selecting the minimum stimulus intensity (in steps of 2% of MSO) that resulted in MEPs > 50 μV in the peak-to-peak amplitude in at least 5 out of 10 consecutive trials (Ziemann et al., 1996; Groppa et al., 2012).

To test for differences in corticospinal excitability, we acquired a MEP IO curve at the ECU hotspot. The intensities for the MEP IO curve were calculated with the estimated electrical field of the NBS system at the hotspot at a depth of ∼20 mm (Danner et al., 2008, 2012). For each subject, the starting intensity was set at 90% RMT and increased in steps of 10 V/m to 140%. Ten MEPs were recorded for each intensity step.

In addition, a cortical map representation was acquired at 110% RMT. This map was extended around the hot spot with evenly distributed stimuli until MEPs could no longer be evoked in the forearm muscles. A visual grid (5 mm × 5 mm × 5 mm), predefined in the navigation software, was used for guidance during the mapping procedure. Each grid cell had been stimulated three times, resulting in 12 pulses/cm^2^. The order of grid cells selected for stimulation was randomized across subjects.

Subjects were instructed to keep their muscles relaxed for the duration of all TMS measurements. We inspected the EMG data during oﬄine analysis, discarding any trials containing muscle preactivation. Less than 1% of all trials were rejected due to contamination by muscle activity.

### Data Analysis

Matlab 2012b (MathWorks) functions with custom build codes were used to analyze the data.

### Resting Motor Threshold and Map Parameters

A predefined grid with a cell size of 3 mm × 3 mm was used for mapping, while the coordinates of all stimulation points were captured by the software of the TMS system. The resulting Map area was obtained by counting the number of active grid cells for responses above 50 μV and multiplying them with 3 × 3 (Melgari et al., 2008). We then calculated the following parameters: mean MEP and maximum MEP of the map, number of active grid cells (map area), and CoG. The CoG was defined as the map location denoting the amplitude weighted center of excitability (Siebner and Rothwell, 2003). The CoGx and CoGy were calculated using

(1) $$\text{CoGx}\;\text{=}\;\Sigma (x_{\text{i}}\;\times \;{\text{MEP}}_{\text{i}})\text{/}\Sigma \text{MEP}$$

(2) $$\text{CoGy}\;\text{=}\;\Sigma (y_{\text{i}}\;\times \;{\text{MEP}}_{\text{i}})\text{/}\Sigma \text{MEP}$$

where MEP_i_ represents the MEP amplitude over (*x*_i_, *y*_i_) location in Cartesian coordinate representation. Mean MEP was defined as the mean of all MEP amplitudes obtained across the map. Maximum MEP coordinates defined the site that evoked the maximum MEP amplitude while progressing with the cortical map. A three-way repeated measures ANOVA (rmANOVA) was performed for differences in map parameters (mean MEP, maximum MEP, map area, and CoG) to ascertain the effect of muscles, pulse waveform, and hemisphere. A Box–Cox transformation was applied in case of non-normality of the data, which was tested by using a Lilliefors test. A two-way rmANOVA was performed for differences in RMT to determine the effect of pulse waveform and hemisphere. *Post hoc* testing was performed with a paired sample *t*-test for the map parameters with a confidence interval of 95% (α = 0.05).

### Input–Output Curve for MEP Amplitude

We fitted a three-parameter Boltzmann sigmoidal function to the MEP IO curve of all subjects for the three muscles. Peak-to-peak amplitude was calculated using Eq. 3 (Devanne et al., 1997).

(3) $$\text{MEP}(\text{S})\;\text{=}\;{\text{MEP}}_{\text{max}}\text{/}\{\text{1}\;\text{+}\;\text{exp}[k(S_{\text{50}}\;\text{-}\;S)]\}$$

In Eq. 3, MEP(S) represents the mean peak-to-peak MEP curve elicited by a stimulus *S* and normalized to the RMT stimulation intensity. The saturation amplitude of the peak-to-peak MEP amplitude is represented by MEP_max_. *S*_50_ stands for the stimulation intensity required to gain 50% of the maximum response, while *k* is the slope parameter of MEP(S), representing the recruitment gain in the corticospinal pathway (Devanne et al., 1997) or trans-synaptic excitability (Ridding and Rothwell, 1997).

This resulted in one mean stimulus–response curve for each of the three muscles in all subjects. A three-way rmANOVA was performed for differences in MEP peak-to-peak amplitude parameters (MEP_max_, *S*_50_, *k*), for the effect of muscles, pulse waveform, and hemisphere. *Post hoc* testing was carried out with a paired sample *t*-test for the parameters of the stimulus–response curve with a confidence interval of 95% (α = 0.05).

The association between muscles was calculated by means of cortical overlapping of each pair of muscles as described in Melgari et al. (2008). Cortical overlap between two muscles is calculated as the percentage of MEP-activated grid cells common to both muscles with respect to the total MEP activated grid cells in any of the muscle.

$$\begin{array}{c} \text{Overlap between EDC and ECU =}\;\frac{\text{|EDC}\;\cap \;\text{ECU|}}{\text{|EDC}\;\cup \;\text{ECU|}}\\ \text{Overlap between ECU and ECR}\;\text{=}\;\frac{\text{|ECU}\;\cap \;\text{ECR|}}{\text{|ECU}\;\cup \;\text{ECR|}}\\ \text{Overlap between EDC and ECR}\;\text{=}\;\frac{\text{|EDC}\;\cap \;\text{ECR|}}{\text{|EDC}\;\cup \;\text{ECR|}} \end{array}$$

## Results

The findings for the RMT (**Figure 1**), the IO curves of the MEP amplitude (**Figure 2**), MAP area (**Figure 3**), mean MEP (**Figure 4**), max MEP (**Figure 5**), CoG (**Figures 6** and **7**), and for the right and left hemisphere (**Figures 8–13**), are presented separately in the following paragraphs.

**FIGURE 1:**
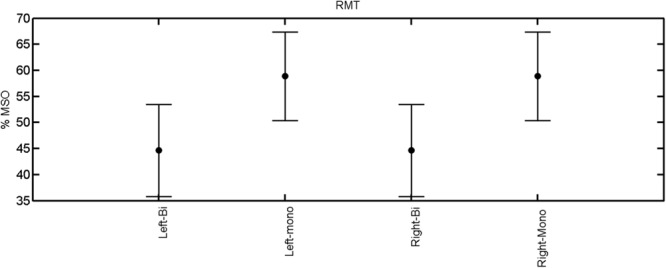
**Resting motor threshold for left and right hemisphere, monophasic and biphasic pulse waveform for all the subjects.** Mean ± SD.

**FIGURE 2:**
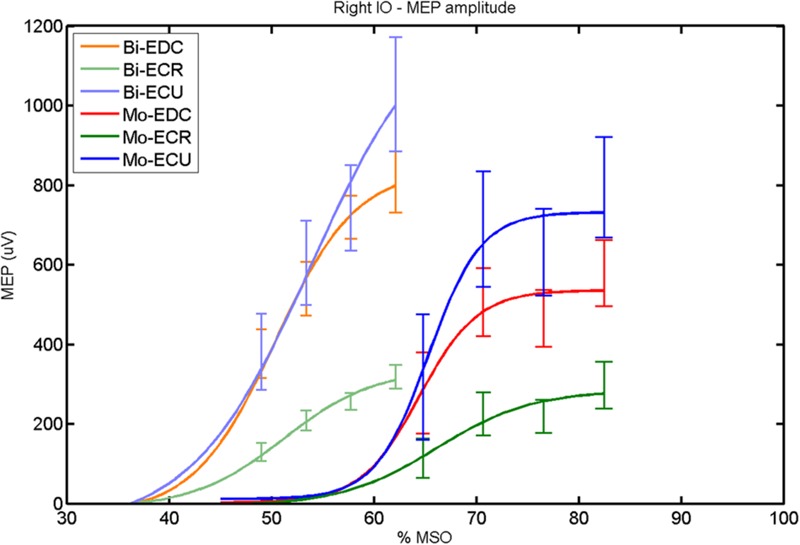
**Right hemisphere: fitted input–output curves for MEP amplitude of the three forearm muscles for both mono- and biphasic stimulation.** Mean ± SD.

**FIGURE 3:**
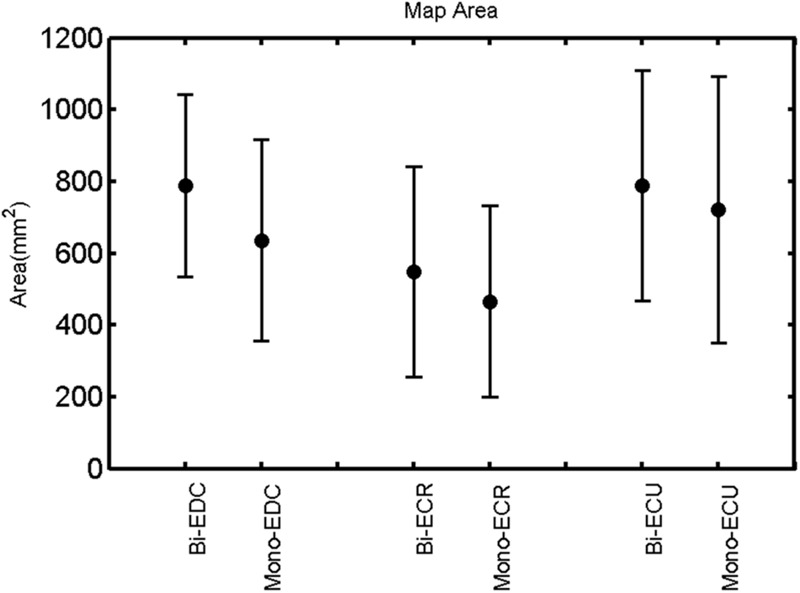
**Right hemisphere: map area of the three forearm muscles for both mono- and biphasic stimulation.** Mean ± SD.

**FIGURE 4:**
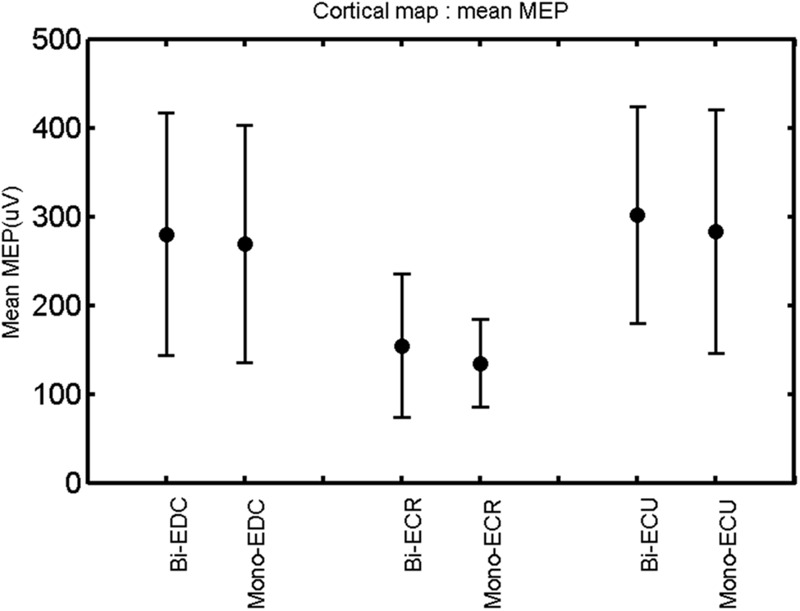
**Right hemisphere: mean MEP of the three forearm muscles for both mono- and biphasic stimulation.** Mean ± SD.

**FIGURE 5:**
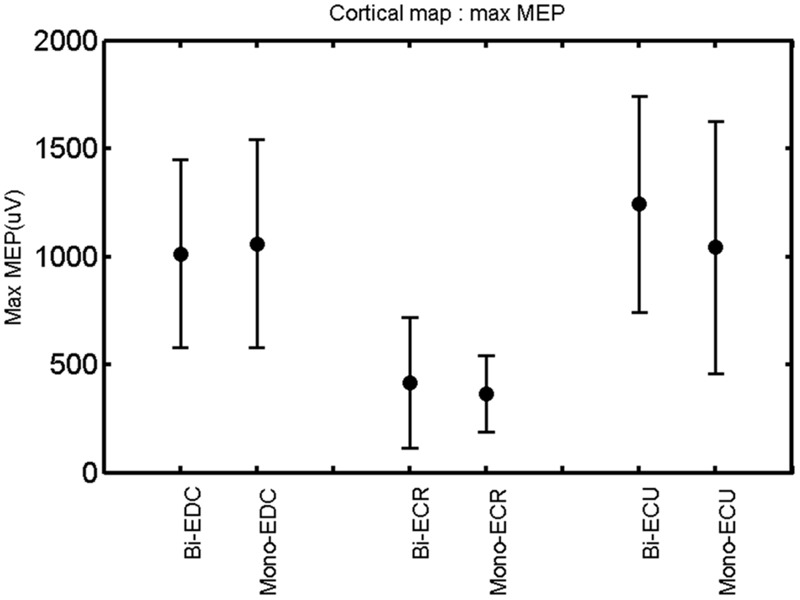
**Right hemisphere: max MEP of the three forearm muscles for both mono- and biphasic stimulation.** Mean ± SD.

**FIGURE 6:**
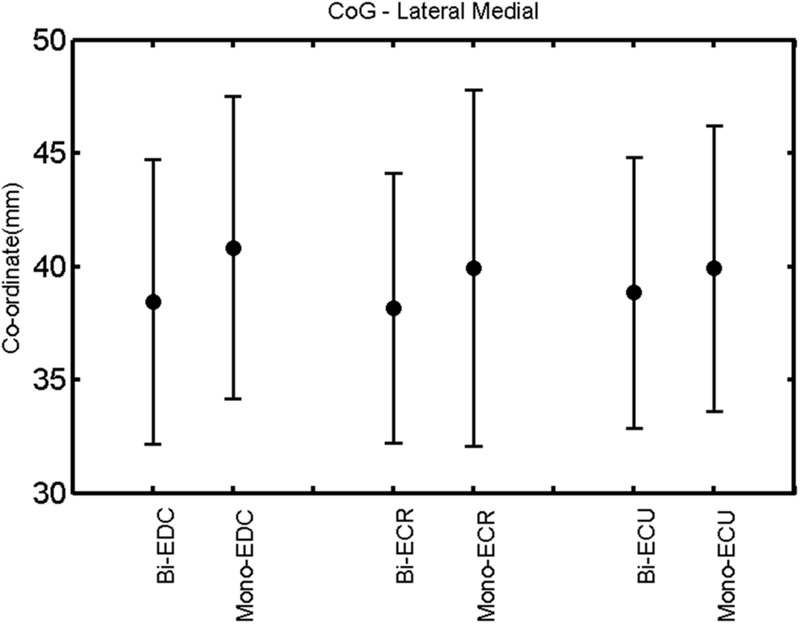
**Right hemisphere: CoG lateral–medial of the three forearm muscles with both mono- and biphasic stimulation.** Mean ± SD.

**FIGURE 7:**
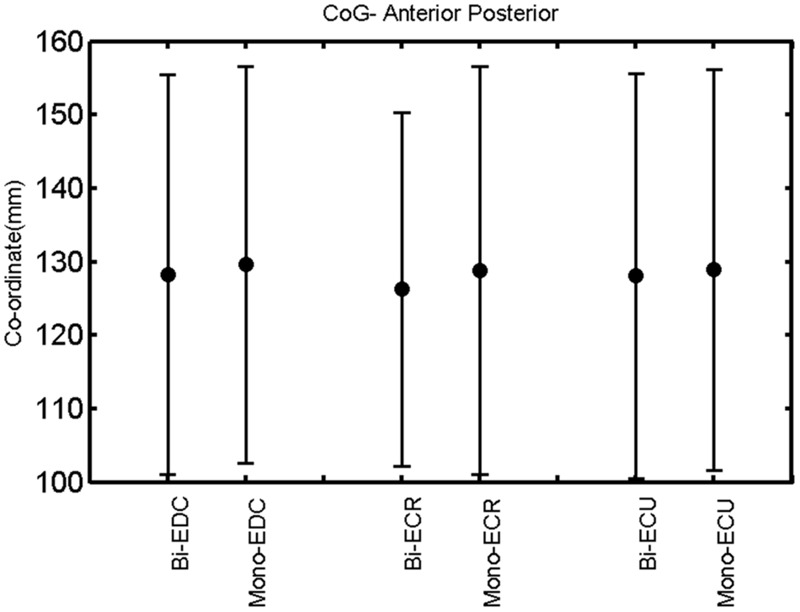
**Right hemisphere: CoG anterior–posterior of the three forearm muscles with both mono- and biphasic stimulation.** Mean ± SD.

**FIGURE 8:**
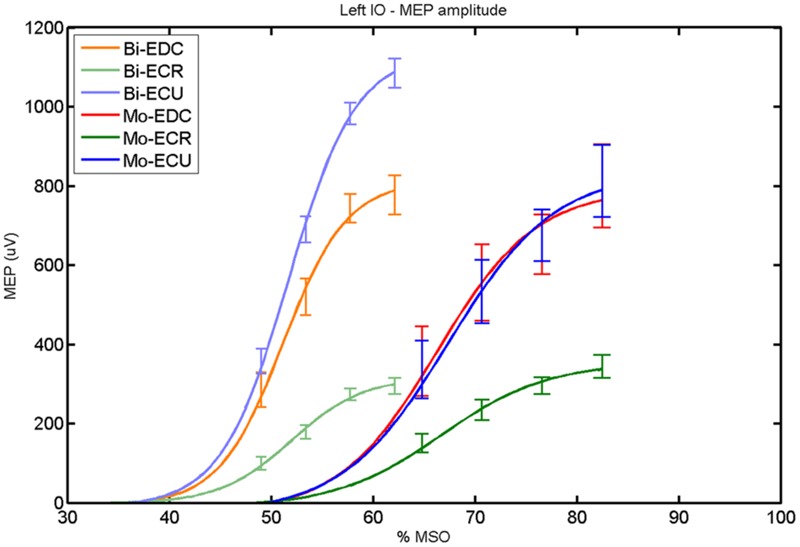
**Left hemisphere: fitted input–output curves for MEP amplitude of the three forearm muscles for both mono and biphasic stimulation.** Mean ± SD.

**FIGURE 9:**
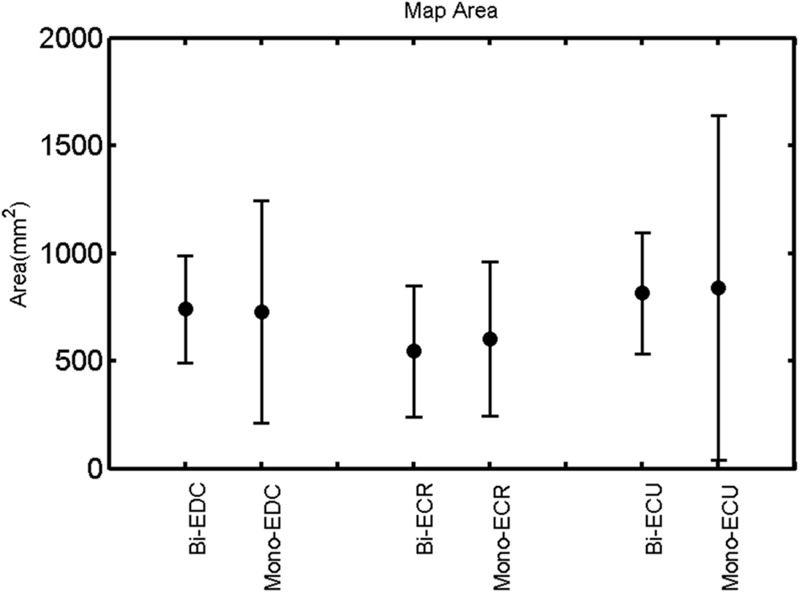
**Left hemisphere: map area of the three forearm muscles for both mono and biphasic stimulation.** Mean ± SD.

**FIGURE 10:**
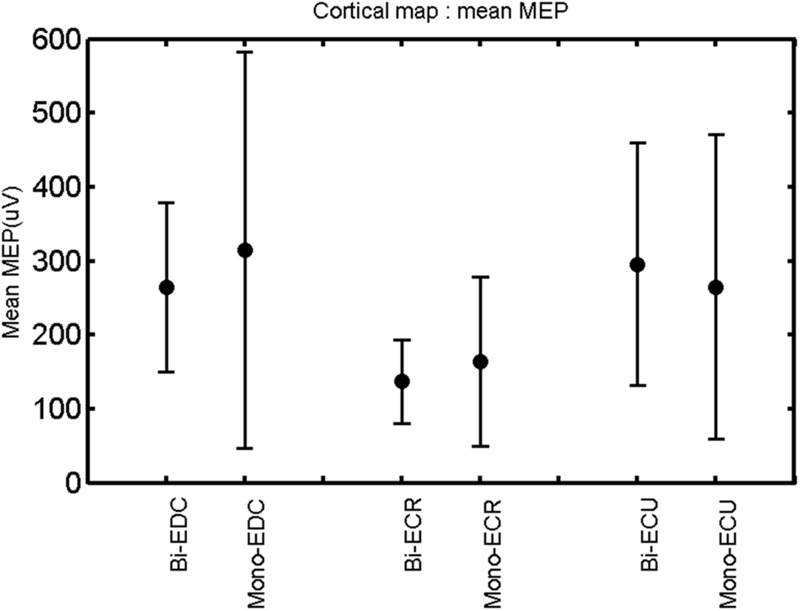
**Left hemisphere: mean MEP of the three forearm muscles for both mono- and biphasic stimulation.** Mean ± SD.

**FIGURE 11:**
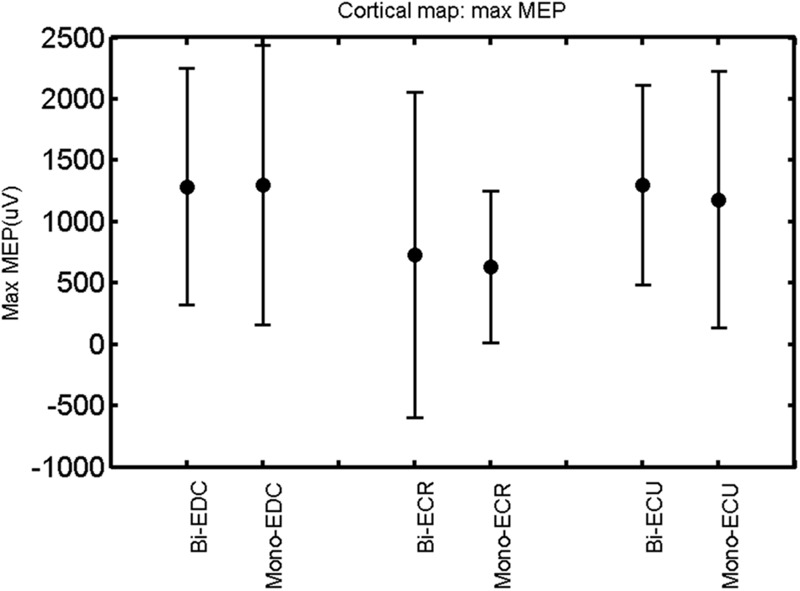
**Left hemisphere: max MEP of the three forearm muscles for both mono- and biphasic stimulation.** Mean ± SD.

**FIGURE 12:**
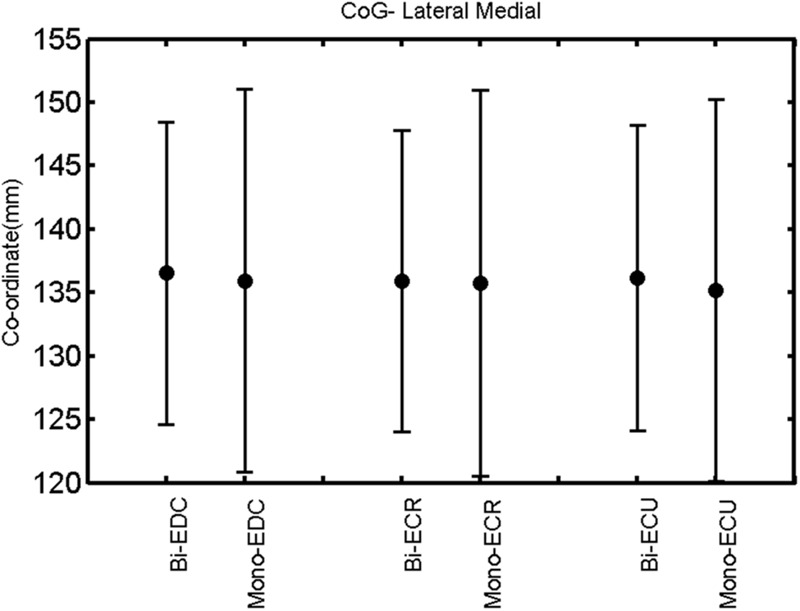
**Left hemisphere: CoG lateral–medial of the three forearm muscles with both mono- and biphasic stimulation.** Mean ± SD.

**FIGURE 13:**
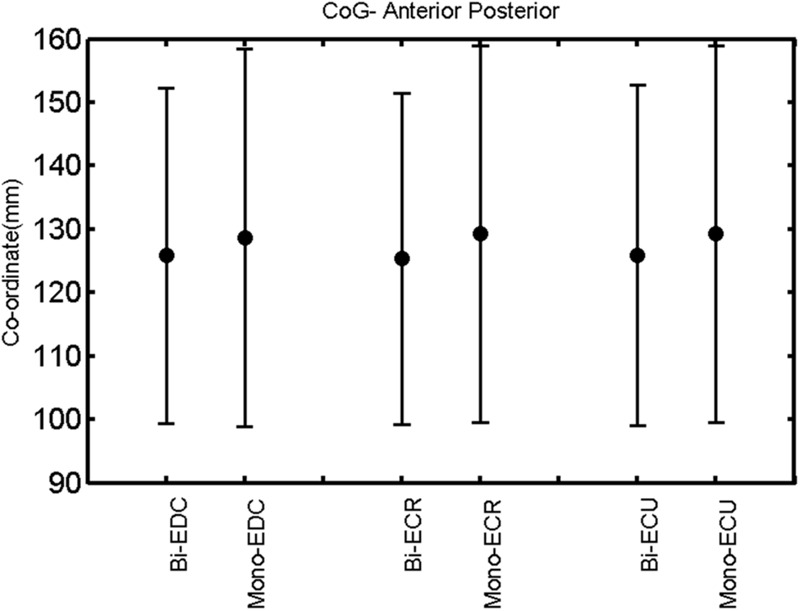
**Left hemisphere: CoG anterior–posterior of the three forearm muscles with both mono- and biphasic stimulation.** Mean ± SD.

### MEP Mapping and RMT

The rmANOVA revealed a significant effect of muscle on map area [*F*_(2,20)_ = 12.56, *p* < 0.001], mean MEP [*F*_(2,20)_ = 22.55, *p* < 0.001], max MEP [*F*_(2,20)_, *p* < 0.001]. There was a significant effect of pulse wave form on the RMT [*F*_(1,8)_ = 7.44, *p* = 0.0259; **Figure 1**].

A *post hoc* paired sample *t*-test between biphasic and monophasic pulse waveforms for the RMT of the same muscle revealed a significant difference for all muscles in both hemispheres [*t*_(18)_ = -3.64, CI = -22.46 to -6.04, *p* = 0.0018] for the RMT. For the left hemisphere, the *post hoc* test between muscles revealed a significant difference for the map area between ECR and ECU [*t*_(20)_ = -2.15; CI = -528.00 to -8.72; *p* = 0.044] and for the mean MEP between ECR and EDC [*t*_(20)_ = -3.31; CI = -207.65 to -47.35; *p* = 0.004], ECR and ECU [*t*_(20)_ = -3.02; CI = 267.21 to -49.24; *p* = 0.007], respectively, for the biphasic pulse form (**Figure 5**). For the right hemisphere, the mean MEP differed significantly between EDC and ECR [bi: *t*_(20)_ = 2.62; CI = 25.63–225.20; *p* = 0.016; mono: *t*_(20)_ = 3.12; CI = 44.80–224.21; *p* = 0.005], ECR and ECU [bi: *t*_(20)_ = -3.34; CI = -239.41 to -55.66; *p* = 0.003; mono: *t*_(20)_ = -3.36; CI = -240.14 to -56.37; *p* = 0.003]. Maximum MEP differed significantly between EDC and ECR [bi: *t*_(20)_ = 3.73; CI = 263.50–928.40; *p* = 0.001; mono: *t*_(20)_ = -4.49; CI = 372.00–1017.10; *p* < 0.001], ECR and ECU [bi: *t*_(20)_ = -4.69; CI = -1194.00 to -459.20; *p* < 0.001; mono: *t*(_20)_ = -3.68; CI = -1059.60 to -293.60; *p* = 0.002], for the biphasic and monophasic pulse form, respectively (**Figure 3**). The CoG revealed no differences between the muscles in either hemisphere (**Figures 4** and **7**).

**Table 1** shows the mean percentage overlap between the three forearm muscles. Cortical overlap between EDC and ECU was higher than that between ECR and ECU and EDC and ECR in both hemispheres. For all combinations of muscles, the cortical map overlap using the monophasic pulse form was larger in the left hemisphere than the maps acquired with the biphasic pulse form, whereas in the right hemisphere, this was observed for EDC–ECR muscle combination only.

**Table 1:** Mean percentage of cortical overlap between pairs of muscles for biphasic and monophasic stimulation maps for the left and right hemispheres.

	Left hemisphere		Right hemisphere	
	Biphasic	Monophasic	Biphasic	Monophasic
EDC–ECU	85.84 ± 3.40	89.39 ± 3.50	87.03 ± 2.80	85.18 ± 4.35
ECU–ECR	72.72 ± 4.50	74.02 ± 4.0	77.23 ± 3.80	73.67 ± 3.74
EDC–ECR	76.37 ± 2.02	81.83 ± 3.75	76.95 ± 3.98	82.11 ± 3.42

*Mean ± SE.*

### MEP IO Curves

With regard to the MEP peak-to-peak amplitude parameters, rmANOVA revealed significant effects of muscle [*F*_(2,16)_ = 10.23, *p* = 0.001], phase [*F*_(1,8)_ = 8.87, *p* = 0.018], and of the interaction of pulse waveform × muscle [*F*_(2,16)_ = 10.26, *p* = 0.001].

In the right hemisphere (**Figure 2**), a *post hoc* paired sample *t*-test revealed a significant difference between ECR and ECU [bi: *t*_(16)_ = -7.25; CI = -686.30 to -376.04; *p* < 0.0001; mono: *t*_(16)_ = -10.58; CI = -547.73 to -364.98; *p* < 0.001] and between ECR and EDC [(bi: *t*_(16)_ = 10.33; CI = -524.73 to -324.52; *p* < 0.001; mono: *t*_(16)_ = -8.77; CI = -334.22 to -204.18; *p* < 0.001] for MEP_max_ for both pulse waveforms. A significant difference was also found between ECU and EDC in MEP_max_ only for monophasic [*t*_(16)_ = 4.06; CI = 89.45–284.85; *p* < 0.001], but not for biphasic stimulation. No significant differences were observed for *S*_50_ and *k*. Moreover, the MEP_max_ differed significantly between biphasic and monophasic stimulation for EDC [*t*_(16)_ = 3.89; CI = 82.27–279.26; *p* = 0.001]. Also among pulse wave forms, *S*_50_ was significantly different for EDC [*t*_(4)_ = -7.48; CI = -19.29 to -8.85; *p* = 0.002] and ECR [*t*_(4)_ = -4.81; CI = -23.56 to -6.32; *p* = 0.009].

For both pulse waveforms (**Figure 8**), a *post hoc* paired sample *t*-test revealed a significant difference for MEP_max_ in the left hemisphere between ECR and ECU [bi: *t*_(16)_ = -11.14; CI = -799.67 to -544.02; *p* < 0.0001; mono: *t*_(16)_ = -10.16; CI = -523.83 to -343.06; *p* < 0.001] and between ECR and EDC [bi: *t*_(16)_ = -8.17; CI = -483.71 to -284.53; *p* < 0.001; mono: *t*_(16)_ = -8.61; CI = -474.26 to -286.98; *p* < 0.001]. A comparison between ECU and EDC showed a significant difference in MEP_max_ [*t*_(16)_ = -3.42; CI = -386.15 to -90.64; *p* = 0.004] for biphasic, but not for monophasic, stimulation. Moreover, the MEP_max_ was significantly higher for biphasic than for monophasic stimulation for ECU [*t*_(16)_ = -3.36; CI = -398.53 to -90.68; *p* = 0.004]. Significant differences in *S*_50_ were also observed among pulse wave forms for EDC [*t*_(4)_ = -7.65; CI = -20.60 to -9.63; *p* = 0.002], ECR [*t*_(4)_ = -9.88; CI = -18.87 to -10.59; *p* < 0.001] and ECU [*t*_(4)_ = -8.85; CI = -20.60 to -10.76; *p* < 0.001].

An interhemispheric pairwise comparison of muscles and pulse waveforms revealed a difference only for the EDC muscle and monophasic stimulation with significantly higher MEP_max_ [*t*_(16)_ = 3.33; CI = 57.83–260.36; *p* = 0.004] for the left hemisphere.

## Discussion

We applied TMS to probe functional synergies of forearm muscles (EDC, ECU, ECR) by harnessing both the convergence and divergence of the corticospinal output; all three muscles are innervated by the nervus radialis which is originating from the ventral roots of the cervical spine (C6–C8). The EDC performs dorsal extension of fingers and wrist, the ECR carries out dorsal extension and radial abduction of the wrist, and the ECU realizes dorsal extension and ulnar abduction of the wrist.

### Cortical Map Overlap

When stimulating the motor cortex at *different spots*, thus utilizing the convergence of the motor system, we captured the functional associations between pairs of muscles by the degree of overlap of cortical motor maps (Melgari et al., 2008). In both hemispheres, we observed a larger overlap, i.e., functional coupling, between EDC and ECU than between ECR and ECU and EDC and ECR (**Table 1**). This result was paralleled by the findings for the mean of all MEP amplitudes obtained across the map. This mean MEP was not different for EDC–ECU, whereas significant differences could be captured for the ECR–EDC and ECR–ECU in both hemispheres when applying biphasic stimulation (**Figures 4** and **10**). Monophasic stimulation could reproduce these findings for the right, but not for the left hemisphere. These interhemispheric differences were also present in a comparison of the maximum MEP amplitudes while progressing with the cortical map. While max MEP did not differ in the left hemisphere (**Figure 11**), significant differences were found in the right hemisphere between ECR–ECU and EDC–ECR with both mono- and biphasic stimulation (**Figure 5**). The map location denoting the amplitude weighted center of excitability, i.e., the CoG revealed no differences between the muscles in either hemisphere (**Figures 6, 7, 12**, and **13**).

### Input–Output Curves

When stimulating the motor cortex at *one spot* and with different stimulation intensities (Devanne et al., 1997), we were able to acquire simultaneous IO curves for EDC, ECU, and ECR, thus utilizing the divergence of the motor system. This was possible due to the vicinity of the cortical representation of the three functionally coupled muscles (**Figures 6, 7, 12**, and **13**). The degree of synergy between pairs of forearm muscles, as suggested by the cortical motor maps, was also captured by the respective IO curves, which were, notably, influenced by the pulse waveform. Monophasic and biphasic stimulation were particularly suitable for disentangling synergistic muscles in the right and left hemisphere, respectively. More specifically, the IO curve of the ECR differed significantly from the IO curve of both EDC and ECR in both hemispheres independent of the pulse waveform applied. This confirms the stronger functional coupling of EDC–ECU, which was already suggested by the cortical map (**Figures 2** and **8**). With regard to the relation between the EDC and the ECU, however, relevant hemispheric differences were found. Significant differences in the MEP_max_ between these two muscles could be captured in the left hemisphere with biphasic stimulation only (**Figure 8**) and in the right hemisphere with monophasic stimulation only (**Figure 2**). Notably, monophasic stimulation of the right (but not left) hemisphere could also disentangle mean MEP/max MEP of the cortical map for ECR–EDC and ECR–ECU, whereas biphasic stimulation was required for this purpose in the left hemisphere.

### Muscle Synergies

Both the cortical maps and the IO curves revealed that ECU and EDC displayed a more pronounced synergy than ECR in both hemispheres. However, only the IO curve demonstrated that this synergy was more prominent in the left than in the right hemisphere and that this functional coupling was mediated via the increased excitability of the EDC in the left hemisphere during monophasic stimulation (**Figure 8**). Consistently, an interhemispheric pairwise comparison of muscles and pulse waveforms revealed a significant difference for the EDC muscle and monophasic stimulation only, with significantly lower MEP_max_ for the right non-dominant hemisphere. More specifically, biphasic stimulation of both hemispheres and monophasic stimulation of the left hemisphere achieved the same MEP_max_ for the EDC (i.e., around 800 μV), whereas monophasic stimulation of the right hemisphere reached a significantly reduced MEP_max_ (i.e., around 500 μV) for the EDC but for none of the other muscles. These interhemispheric differences suggest that the lower preference of the left hand, i.e., finger extensors, during activities of daily living in the group of right-handers examined in this study might be indexed by less excitability of the neural circuits in the right, non-dominant hemisphere, which are primarily addressed by monophasic stimulation at higher intensities (**Figure 2**).

However, it should be considered that other factors than those mentioned here may have influenced the IO curves as well. More specifically, different muscles may be characterized by different muscle mass and amount of activating corticospinal neurons, which will then affect the response to stimulation and the thresholds for excitability. Controlling for such intrinsic differences was not possible with the current approach but might be considered in future studies.

### Motor Cortex Circuitry

In this context, stimulation intensity, pulse wave form, and current direction may provide distinct information on the underlying motor cortex circuitry: TMS over M1 evokes multiple descending volleys, generated by direct (D wave) and indirect (I waves), i.e., via presynaptic neurons, and/or activation of pyramidal tract neurons (Di Lazzaro et al., 2001a). At higher amplitudes, MEPs are believed to reflect the recruitment of such additional presynaptic neuronal pools, e.g., later I waves (Devanne et al., 1997; Di Lazzaro et al., 2001a). Moreover, biphasic pulses are thought to activate a larger set of intracortical neurons than monophasic stimulation (Di Lazzaro et al., 2001b), enabling us to probe their common modulation in muscle synergies. At the same time, biphasic rather than monophasic stimulation enables MEPs to be invoked with less energy injection, thereby improving focality and sensitivity (Raffin et al., 2015). When applied at the same intensity, i.e., stimulator output, biphasic stimulation is more powerful in inducing a systematic shift toward a lower motor threshold than monophasic stimulation (Kammer et al., 2001), as verified in the present study for all three forearm muscles.

In this study, the orientation of the induced current of the stimulus in the brain was posterior–anterior for the monophasic waveform, and posterior–anterior in the first phase and anterior–posterior in the second phase of the biphasic waveform; the effect seems to be stronger for the second than the first phase (Kammer et al., 2001). The differences observed in this study might therefore also be related to different current directions, which may depolarize different sets of cortical interneurons with different thresholds. Epidural recordings of corticospinal volleys suggested that early I waves are preferentially activated by anteriorly oriented pulses at low thresholds and that later I waves are activated with posteriorly oriented pulses at higher thresholds (Di Lazzaro et al., 2001a). On the basis of these and other observations, it has been proposed that different structures are activated by different waveforms and current directions or that the same sets of interneurons in the motor cortex are activated at different sites (Sommer et al., 2006). IO curves and pulse waveforms, i.e., monophasic or biphasic, may therefore both provide complementary information with regard to recruitment patterns of corticospinal excitability without providing conclusive knowledge about the underlying neural circuit dynamics (Devanne et al., 1997; Di Lazzaro et al., 2001a,b).

### Differences Between the Hemispheres

Interhemispheric differences of corticospinal excitability in rest have already been probed with TMS, and have revealed inconsistent findings. With regard to the motor threshold and/or the MEP amplitude, no differences between hemispheres were found for musculus biceps brachii (Macdonell et al., 1991), the musculus abductor pollicis brevis (Triggs et al., 1999), musculus flexor carpi radialis (Triggs et al., 1999), and the musculus abductor digiti minimi (Cicinelli et al., 1997). However, one study found a difference in the musculus abductor digiti minimi (Macdonell et al., 1991). Notably, these studies did not acquire a complete IO curve nor did they apply different pulse wave forms. In the present study also, only one of the three examined forearm muscles revealed interhemispheric differences of corticospinal excitability. Notably, an asymmetry in cortical motor representation could even be shown in studies in which no interhemispheric differences of motor threshold and/or the MEP amplitude was found (Triggs et al., 1999). The cortical motor map might therefore be more suitable for detecting differences between hemispheres, particularly when applying refined techniques that align the TMS coil on the basis of the individual shape of the central sulcus so as to capture the somatotopy in the primary motor hand area (Raffin et al., 2015). However, these approaches might still not be precise enough to distinguish between the natural daily or weekly fluctuations of the motor map extent (Weiss et al., 2013) and lasting cortical plasticity due to hemispheric dominance. Such a differentiation would necessitate stable cortical map parameters that are resistant to such natural fluctuations. A projection, interpolation, and coregistration technique for estimating nTMS sites onto the individual anatomy (Kraus and Gharabaghi, 2015), i.e., following the surface curvature of gyri and sulci, was recently proposed which might overcome these limitations (Kraus and Gharabaghi, under review) as well as capture muscle-specific modulations of corticospinal excitability (Kraus et al., 2016).

## Conclusion

The findings of the present study indicate the feasibility of probing corticospinal recruitment patterns and functional synergies with TMS. Independent of the pulse wave form applied, the CoG were similar across muscles for both hemispheres. This allowed recording combined IO curves for these muscles with different pulse waveforms, thereby providing complementary information on neural circuit dynamics underlying synergistic muscles and their potential neuroplastic modulation. More specifically, monophasic but not biphasic stimulation resulted in a significant interhemispheric modulation of the EDC excitability and of IO curve similarity with the ECU, indicating stronger EDC–ECU synergy for the left than for the right hemisphere. While the direction of this lateralization is most likely linked to the right-handedness of the examined subjects, we interpret the waveform specificity as an indicator of different interneuronal recruitment. The increased intersubject variance of the map area in the left hemisphere without interhemispheric differences of the mean MEP or the CoG, suggests a cortical reorganization pattern with mosaic-like topographies, a hypothesis that needs empirical verification in further mapping studies.

## Author Contributions

JM analyzed data and edited the paper. AK designed and performed research. RB analyzed data and edited the paper. AG designed research, analyzed data, and wrote the paper.

## Conflict of Interest Statement

The authors declare that the research was conducted in the absence of any commercial or financial relationships that could be construed as a potential conflict of interest.
